# Patient-Reported Outcomes for Quality of Life Assessment in Atrial Fibrillation: A Systematic Review of Measurement Properties

**DOI:** 10.1371/journal.pone.0165790

**Published:** 2016-11-01

**Authors:** Dipak Kotecha, Amar Ahmed, Melanie Calvert, Mauro Lencioni, Caroline B. Terwee, Deirdre A. Lane

**Affiliations:** 1 Institute of Cardiovascular Sciences, University of Birmingham, Birmingham, United Kingdom; 2 City Hospital, Sandwell and West Birmingham Hospitals NHS Trust, Birmingham, United Kingdom; 3 Institute of Applied Health Research, University of Birmingham, Birmingham, United Kingdom; 4 University Hospitals Birmingham NHS Trust, Birmingham, United Kingdom; 5 Department of Epidemiology and Biostatistics and the EMGO Institute of Health and Care Research, VU University Medical Center, Amsterdam, The Netherlands; University of Glasgow, UNITED KINGDOM

## Abstract

**Background:**

Atrial fibrillation is a large and growing burden across all types of healthcare. Both incidence and prevalence are expected to double in the next 20 years, with huge impact on hospital admissions, costs and patient quality of life. Patient wellbeing determines the management strategy for atrial fibrillation, including the use of rhythm control therapy and the clinical success of heart rate control. Hence, evaluation of quality of life is an emerging and important part of the assessment of patients with atrial fibrillation. Although a number of questionnaires to assess quality of life in atrial fibrillation are available, a comprehensive overview of their measurement properties is lacking.

**Methods and Results:**

We performed a systematic review of the measurement properties of atrial fibrillation-specific health-related quality of life questionnaires. Methodological quality was assessed using the Consensus based Standards for selection of health Measurement Instruments (COSMIN) checklist, with measurement properties rated for quality against optimal criteria and levels of evidence. We screened 2,216 articles, of which eight articles describing five questionnaires were eligible for inclusion: Atrial Fibrillation 6 (AF6), Atrial Fibrillation Effect on QualiTy-of-Life (AFEQT), Atrial Fibrillation Quality of Life Questionnaire (AFQLQ), Atrial Fibrillation Quality of Life (AFQoL), and Quality of Life in Atrial Fibrillation (QLAF). Good reliability (internal consistency and test-retest reliability) was demonstrated for AF6, AFEQT, AFQLQ and AFQoL. Content, construct and criterion validity were positively rated only in AFEQT. Responsiveness was positively rated only in AFEQT, but with limited evidence. Overall, AFEQT showed strong positive evidence for 2 of 9 measurement properties, compared to one for AFQoL and none for the remaining questionnaires.

**Interpretation:**

Given the low ratings for many measurement properties, no single questionnaire can be recommended, although AFEQT performed strongest. Further studies to robustly assess reliability, validity and responsiveness of AF-specific quality of life questionnaires are required. This review consolidates the current evidence for quality of life assessment in patients with atrial fibrillation and identifies priority areas for future research.

## Introduction

Atrial fibrillation (AF) is the most common cardiac arrhythmia and is predicted to double in prevalence over the next 20 years. It is not only associated with adverse prognosis, but accounts for considerable healthcare expenditure. [1] Numerous studies have identified a substantial reduction in health-related quality of life (QoL) in AF populations. [2] Patients with AF experience symptoms such as palpitations, exercise intolerance, dizziness and dyspnea, which frequently limit capacity to undertake daily activities. [3, 4]

Apart from anticoagulation to prevent strokes, current management of AF is focused on reducing symptoms and improving QoL. [5] Even well-established therapies in AF have proven to have little impact on death and other adverse outcomes, including rate and rhythm control therapy. [6–9] Although the fundamental reason for pursing such therapies is to improve QoL, there is limited appreciation of how to measure QoL in clinical practice.

A variety of both generic and AF-specific questionnaires are available to assess patient-reported QoL. [10] Generic patient reported outcome measures (PROMs), such as the SF-36 and EQ-5D questionnaires, have the advantage of allowing comparison of QoL in patients with different diseases, but are less sensitive to the effects of a single disease on QoL. [11] With the growing burden of AF, the relatively high cost of treatment, and the appreciation of QoL as a treatment objective, there has been substantial interest in the development of AF-specific PROMs for use in both clinical research and routine practice. However, these have yet to enter routine clinical practice, in part due to concerns about their validity and responsiveness. [12]

Our objective was to perform a systematic review of the measurement properties of disease-specific PROMs measuring QoL in AF, assessing studies which designed and validated the questionnaires. We aim to provide clinicians with an understanding of whether these instruments would likely be of value in research and clinical practice. We utilized a comprehensive methodology from the Consensus based Standards for selection of health Measurement Instruments (COSMIN) group [13], which includes rigorous assessment of validity, reliability, and responsiveness of QoL questionnaires.

## Methods

### Eligibility criteria and search strategy

We included all studies that examined at least one or more measurement properties of a disease-specific PROM measuring QoL in patients with AF (aged 18 years and older). We permitted inclusion of studies with interviewer-based administration, as long as questionnaires were self-reported by patients. Studies were required to be published as a full-text article. Articles were excluded if the article failed to specify the type of tool utilized. There was no restriction on language, date of publication or type of AF.

A systematic search was performed in Medline, EMBASE, PsycINFO and CINAHL from inception until the 15^th^ February 2015 and in the Cochrane library from 1993 to 9^th^ March 2015. The search strategy (see S1 Appendix) consisted of 3 filters composed of search terms for the following: (1) AF; (2) PROMs; and (3) measurement properties. The latter two filters were developed at the University of Oxford and VU University Medical Center [14] respectively (available at www.cosmin.nl). All filters were adapted for each database. Reference lists of the included studies were also manually searched in addition to articles that cited them.

This review was prospectively registered with the PROSPERO database of systematic reviews (http://www.crd.york.ac.uk/PROSPERO/display_record.asp?ID=CRD42015016600). In this paper, we report on our primary outcome which addresses the quality of AF-specific PROMs using the COSMIN methodology.

### Data collection and synthesis

For each study, two investigators independently extracted and tabulated data on a standardized data extraction form. Discrepancies and missing data were resolved by group discussion, reference to the original publication and additional adjudication. Two studies published in Japanese [15, 16] were first translated by a Cardiologist fluent in both the Japanese and English languages.

The COSMIN checklist was used by two independent reviewers to assess the methodological quality of the included studies. [13] This tool distinguishes three main domains (reliability, validity and responsiveness), subdivided into nine measurement properties. For each measurement property, numerous standards are included in the form of questions to determine the quality of the study. Each item per measurement property is scored as excellent, fair, good or poor, with the lowest rating determining the overall quality score for that study (“worst score counts” method).

Measurement properties were rated for quality against criteria for optimal measurement properties. Each result was rated as either positive (+), negative (-) or indeterminate (?), providing that methodological quality was not poor. [17] Table 1 summarizes the definitions of the nine measurement properties assessed and the preferred methods for their assessment, based on the COSMIN taxonomy, with detailed criteria presented in S1 Table. [18, 19] Best evidence synthesis was performed by applying levels of evidence developed by Terwee *et al*. (www.cosmin.nl), combining the ratings of study quality with the strength of findings to determine an overall judgement for each questionnaire, as demonstrated in S2 Table.

**Table 1:** Definitions of measurement properties.

Measurement Property	Definition	Quality criteria for positive rating *
**1. Reliability**		
Internal Consistency	The extent to which items of a questionnaire are interrelated in their underlying construct	Cronbach’s alpha (+ if ≥0.70)
Test-retest reliability	The proportion of the total variance in the measurements which is due to true differences between patients.	Intra-class correlation coefficient or weighted Kappa (+ if ≥0.7 or Pearson’s r ≥0.8)
Measurement Error	The extent of change not attributable to the true alteration in the patient’s quality of life	Minimal important change (MIC) (+ if MIC > smallest detectable change or MIC outside the limits of agreement)
**2. Validity**		
Content Validity	Assesses whether the questionnaire adequately reflects the construct of interest	Relevance to target population (+ if relevant to the construct measured and comprehensive)
Construct Validity		
Structural Validity	Consistency between factor structure and the underlying construct	Factor analysis (+ if factors explain at least 50% of variance)
Hypothesis testing	Convergent: Determines whether expected similar domains between measurement tools are in fact similar Discriminant: Determines whether expected dissimilar domains between measurement tools are in fact unrelated	Correlation coefficient (+ if ≥0.5 with an instrument measuring the same construct, or ≥75% in accordance with hypotheses and concordance with stated constructs)
Cross-cultural validity	Assesses whether a translated questionnaire adequately reflects the original questionnaire	Comparison of different language versions (+ if original factor structure confirmed or no important differences)
Criterion Validity	Similarity between scores derived through the questionnaire against those from a gold standard	Correlation coefficient with an accepted gold standard measure (+ if ≥0.7)
**3. Responsiveness**		
Response to clinical change	The ability to detect change in quality of life over time	Correlation and hypothesis concordance (+ if correlation ≥0.50 with instrument measuring same construct or ≥75% in accordance with hypotheses or area under the curve ≥0.70)

* For a full description of the criteria for measurement properties, see S1 Table.

## Results

### Study characteristics

**Fig 1:**
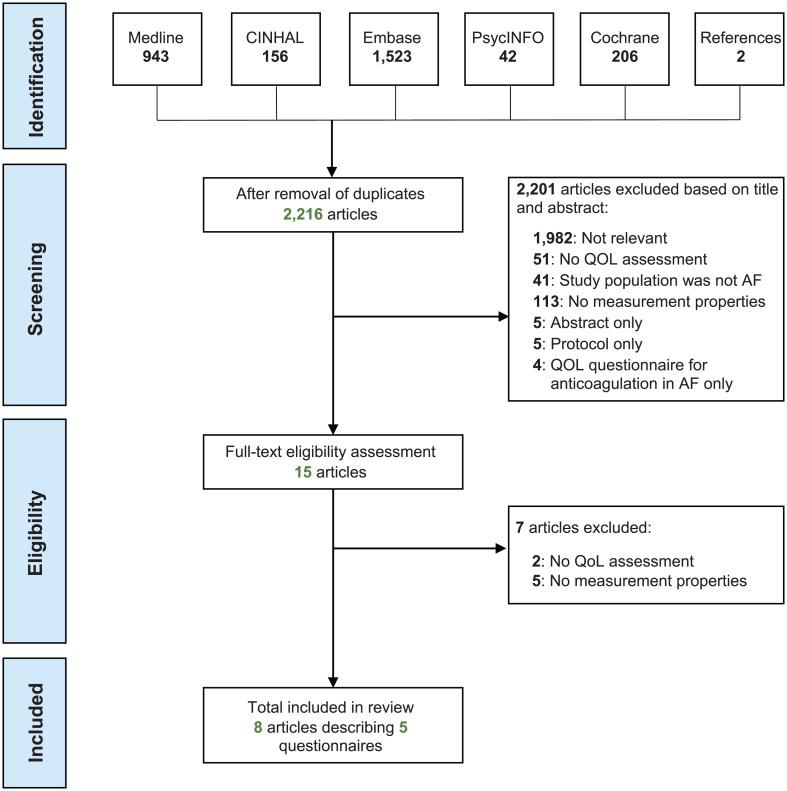
Selection of studies flowchart. Study inclusion flowchart. See S1 Appendix for search strategy.

The results of the search strategy are outlined in Fig 1. After removal of duplicates, the literature search yielded 2,216 articles of which eight were included in the final analysis. [15, 16, 20–25] A summary of studies excluded at the full-text stage is presented in S3 Table.

We identified five questionnaires developed and validated for the assessment of quality of life in AF: Atrial Fibrillation 6 (AF6) [24, 25], Atrial Fibrillation Effect on QualiTy-of-Life (AFEQT) [20], Atrial Fibrillation Quality of Life Questionnaire (AFQLQ) [15, 16], Atrial Fibrillation Quality of Life (AFQoL) [21, 22], and Quality of Life in Atrial Fibrillation (QLAF). [23]

The characteristics of the questionnaires included in this review and the study populations in which their measurement properties were evaluated are summarized in Table 2. The QoL questionnaires were assessed in a variety of geographical locations, however patients were of a similar mean age (62–67 years). Women were under-represented in all of the cohorts (20–43%). The type of AF varied in the individual studies, although on average there was an even split between paroxysmal and more persistent forms.

**Table 2:** Characteristics of included studies.

Instrument	Population characteristics					Questionnaire characteristics		
	Geographical location	Sample size of studies	Mean age (years ± SD)	Women (%)	Type of AF (%)	Number of items	Domains	Response options
**AF6**[24, 25]	Sweden	111	67 ± 12	20%	Not reported	6	Dyspnea at rest, dyspnea on exertion, limitation in daily life due to AF, discomfort due to AF, fatigue due to AF, anxiety due to AF	10 point Likert scale
**AFEQT**[20]	Canada & US	213	62 ± 12	42%	Paroxysmal 66%, persistent 29%, permanent 5%	20	Symptoms, daily activities, treatment concerns, treatment satisfaction	7 point Likert scale
**AFQLQ**[15, 16]	Japan	40 & 172	64 ± 10	24%	Paroxysmal 57%, persistent 43%	26	Type and frequency of symptoms, severity of symptoms, psychological aspects, limitation in daily life	4–6 options of ranging severity dependent on domain
**AFQoL**[21, 22]	Spain	112 & 417	62 ± 12	35%	Paroxysmal 53%, permanent 47%	18	Psychological, physical, sexual activity	5 point Likert scale
**QLAF**[23]	Brazil	63	63 ± 12	43%	Paroxysmal 38%, persistent 32%, permanent 30%	22	Palpitations, chest pain, breathlessness, dizziness, drugs, direct current cardioversion, ablation	Letters assigned to text options and yes/no tick boxes

### QoL instruments

**Fig 2:**
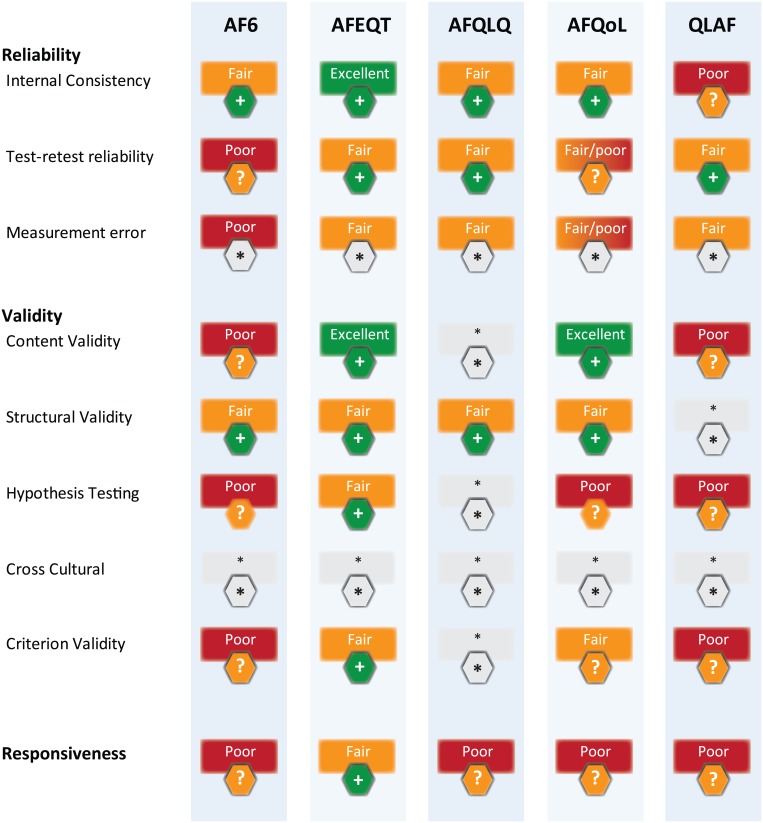
Summary of assessment. For each measurement property, the PROM is assessed for methodological quality of the study (excellent, good, fair or poor) and given an overall rating (positive [+], negative [-] or indeterminate/unknown [?]) for the results. A level of evidence was applied, combining the number and quality of the studies with the strength of findings. Studies that have poor quality are given an ‘indeterminate /unknown’ rating due to the limited level of evidence. For AFQoL, reliability was assessed differently in the two related studies. * Insufficient data were available to rate this criterion. See Table 1 for assessment criteria.

Results of the assessment of validity for each instrument are detailed in S4 Table and for reliability and responsiveness in S5 Table. It was not possible to rate any of the instruments in relation to measurement error, as statistical evaluations were not reported. Similarly, there were no data on cross cultural validity, and hence study quality and instrument ratings were not performed for these domains. A summary of findings is presented in Fig 2. Best evidence synthesis to provide an overall appraisal of each questionnaire is detailed in Table 3, limited by the availability of only one or two published articles for each questionnaire. Below, we concisely review the main results for the individual QoL instruments alphabetically.

**Table 3:** Synthesis of results.

Measurement property	AF6	AFEQT	AFQLQ	AFQoL	QLAF
Internal Consistency	+	+++	+	+	?
Test-retest reliability	?	+/-	+	+/-	+
Measurement error	n/r	n/r	n/r	n/r	n/r
Content Validity	?	+++	n/r	+++	?
Structural Validity	+	+	+	+	n/r
Hypothesis Testing	?	+	n/r	?	?
Cross Cultural	n/r	n/r	n/r	n/r	n/r
Criterion Validity	?	+	n/r	+/-	?
Responsiveness	?	+	?	?	?

“+++” or “- - -” = strong positive or negative evidence; “++” or “- -” = moderate positive or negative evidence; “+” or “-” = limited positive or negative evidence; “+/-” = conflicting findings; “?” = unknown, due to poor methodological quality; n/r = not rated due to insufficient data. See S2 Table for assessment criteria.

#### 1. AF6

AF6 was evaluated in 2 articles, both utilizing the same cohort of 111 patients recruited in Sweden. [24, 25]

Reliability: The quality of the study was fair for internal consistency and reliability, with both of these measurement properties rated positively. Internal consistency was only evaluated in patients who underwent direct current cardioversion (which may not be representative of general AF patients and can variably affect QoL depending on success). Test-retest reliability was only assessed in 9 patients who had failed cardioversion, with 3 of the 6 items (limitations in daily life due to AF, discomfort due to AF and fatigue due to AF) associated with poor coefficient values.

Validity: Content validity and hypothesis testing of this instrument were rated as indeterminate due to poor ratings for reported methodology. For criterion validity, appropriate statistics were not reported. Structural validity assessment was deemed of fair quality overall, although statistical methods and handling of missing data were excellent and the instrument was positively rated for this measurement property.

Responsiveness: Only assessed in a single study of poor methodological quality, which did not describe the construct of the comparator (SF-36) or detail the handling of missing data or the underlying hypotheses. [25]

Synthesis: Two measurement properties was positively rated based on limited evidence.

#### 2. AFEQT

AFEQT was evaluated in a single article composed of 213 patients recruited from hospitals across North America. [20] The population was predominantly of European descent and English-speaking.

Reliability: The quality of the study was excellent for internal consistency and fair for test-retest reliability, due to inadequate information regarding the environment of questionnaire administration. Both measurement properties were rated as positive.

Validity: The quality of the study on content validity was deemed methodologically excellent, whereas the quality of the study regarding structural validity, hypothesis testing and criterion validity were rated fair as the handling of missing data was not detailed. All of these validity aspects were rated positive.

Responsiveness: Although there was excellent description of hypotheses, comparators and methods, the overall quality of the study was rated fair, as responsiveness was only assessed in cohorts of patients who received an intervention (66 patients with medication adjustment and 76 with ablation). It was unclear how missing data were handled, and the expected or achieved correlations were not reported. The responsiveness of the instrument was however, rated as positive (data presented in S5 Table). Comparison with generic QoL measures (SF-36 and EQ-5D) resulted in smaller effect sizes, although the responsiveness of the AFEQT was comparable to the Toronto AF Symptoms Check List and Atrial Fibrillation Severity Scale.

Synthesis: Two measurement properties showed strong positive evidence, and four were positive based on limited evidence.

#### 3. AFQLQ

AFQLQ was studied in two articles, both recruiting patients from Japan. [15, 16]

Reliability: Assessed in a single article, [16] quality was categorized as methodically fair for both internal consistency and test-retest reliability. Although the sample size was >100, both measurement properties were marked down due to inadequate information on missing data and potential differences in test conditions during resampling. Both measurement properties were rated positive.

Validity: Structural validity was investigated in a single article. [15] The study was methodologically fair as assessment was only performed in a small number of patients and the handling of missing data was not clearly reported. The instrument was positively rated for structural validity. No information was available on other validity aspects.

Responsiveness: Assessed in a single article [16], this study was rated as methodically poor. It was unclear if changes occurred in the patients during the interim period, either in treatment or symptoms, and the study failed to detail expected hypotheses.

Synthesis: Three positive measurement properties with limited evidence, however there was insufficient data to rate 5 out of 9 measurement properties.

#### 4. AFQoL

AFQoL was assessed in two articles recruiting Spanish patients from hospitals across Madrid and Bilbao. [21, 22]

Reliability: An evaluation of internal consistency was rated as methodologically fair in both articles, whereas test-retest was rated fair in one [21] and poor in the other [22], due to the relatively small sample size, uncertainty if the test conditions were the same, and lack of details regarding the handling of missing data. The instrument was rated positive for internal consistency in both articles [21, 22], but indeterminate for test-retest reliability.

Validity: Content validity and structural validity were assessed in a single article. [22] The quality of content validity assessment was methodologically excellent, while structural validity was deemed fair due to omission of details on the handling of missing data. The instrument was rated positively for both of these measurement properties. The study on hypothesis testing was methodologically poor due to a lack of detail on the self-perceived health status scale employed, hence the instrument was rated indeterminate. Criterion validity was assessed in a single article [21], with fair study quality but an indeterminate rating as QoL was compared with patients post-myocardial infarction.

Responsiveness: Assessed in a single article of poor methodologically quality [21], as only correlations between baseline SF-36 and AFQoL were reported.

Synthesis: One measurement property showed strong positive evidence, and two were positive based on limited evidence.

#### 5. QLAF

QLAF was assessed in a single article consisting of 63 patients recruited from centers across Brazil. [23]

Reliability: The assessment of internal consistency was rated as methodically poor as factor analysis was not performed. Study quality for test-retest reliability was fair, due to the small sample size and insufficient detail on the handling of missing items. The instrument was rated as positive for this measurement property.

Validity: Content validity was rated as methodologically poor, as the questionnaire was administered to a small target population and there was no patient involvement in the questionnaire design. Prior hypotheses regarding expected correlations between the SF-36 and QLAF were not stated when undertaking hypothesis testing and appropriate statistics were not used during criterion validity assessment; thus both construct and criterion validity were rated as methodologically poor.

Responsiveness: Poor study quality due to a lack of appropriate statistical analysis.

Synthesis: One measurement property showed positive evidence based on limited evidence.

## Discussion

This comprehensive systematic review identified five health-related QoL questionnaires used to assess AF populations, and evaluated their measurement properties using the COSMIN approach. To our knowledge, this is the first study to systematically evaluate the methodological quality of studies and the measurement properties of QoL questionnaires in AF patients. Our key finding was the lack of robust psychometric testing of any of the currently available AF-specific QoL instrument, particularly for measurement error. We found deficiencies in most instruments in relation to reliability, construct and criterion validity, but particularly in the responsiveness of questionnaires. The results raise important questions about the use of these instruments in research and daily clinical practice, and suggest the need for further evaluation before implementation.

Quality of life encompasses a person's perceptions of their physical and psychological state, social relationships and environment. [26] Many generic and disease-specific questionnaires exist to assess QoL in AF patients. The Short Form 36 (SF-36) is perhaps the most common generic QoL measure [4], and often used as a gold-standard comparator in the validation of other questionnaires (although as there is no ‘true’ gold-standard measure, criterion validity remains problematic to adequately appraise). Generic measures are useful to determine overall QoL in the population, particularly to quantify health utility or for comparing between diseases and populations. However, they may not be as sensitive to the effect of a single disease on QoL [11], whereas disease-specific questionnaires assess domains more relevant to the particular condition and may provide more targeted information to inform shared-decision making. [10] There is increasing interest in evaluating the effect of management strategies on QoL among AF patients, but for clinical confidence, the tool used should demonstrate good responsiveness, in addition to being a reliable and valid measure. Indeed, a recently developed QoL questionnaire for patients undergoing catheter ablation for cardiac arrhythmias has demonstrated responsiveness to clinical change. [27]

This review utilized the COSMIN approach for performing the systematic review. The COSMIN taxonomy was developed after an international Delphi study [13], using a consensus of terminology across validation studies and allowing standardized objective assessment of measurement properties. Further, the group have developed standards (the COSMIN checklist) for how measurement properties should be evaluated [28] and provide a structured protocol for undertaking such reviews to a high quality (http://www.cosmin.nl/). The COSMIN methodology has been incorporated into several other reviews to evaluate PROM assessment across a variety of diseases, including chronic obstructive pulmonary disease [29], osteoarthritis [30], cancer [31], multiple sclerosis [32] and Parkinson’s Disease. [32] Whilst the standards within COSMIN are high, the thresholds for reliability are in line with the International Society for Quality of Life Research (ISOQOL) requirements. [33] It could be argued that high standards are needed to drive improvements in the methodology of development and validation of PROMs, and allow clinicians to have greater confidence in their use. As with any system based on scientific judgment, our results based on the COSMIN approach are only one component of evaluating quality of life questionnaires.

A specific limitation of our review was the inclusion of questionnaires developed and validated before the COSMIN checklist became widely available, which may have impacted on the scores assigned for methodological quality. This may be less of an issue in future reviews, where validation studies would ideally have incorporated the COSMIN checklist. There are other methodologies available for evaluating PROMs, for example the Federal Drug Administration scheme, which utilizes broadly similar assessment categories. [34] However, the COSMIN approach offers a more quantifiable appraisal of measurement properties. Secondly, the inclusion of two Japanese papers requiring translation meant these articles were assessed by a sole reviewer, which may impact on internal validity. We are also limited to the ‘general’ AF populations studied, whereas patients with specific comorbidities may respond differently to PROMs. For example, heart failure is a common comorbidity in AF [35] that is underrepresented in the studies evaluated.

The content of these questionnaires demonstrates some similarities among them, with 4 out of 5 examining the type and/or frequency of symptoms (the only exception being AFQoL), and all except QLAF assessing the impact of AF on daily activities, physical and psychological functioning, albeit to differing extent. Synthesis of results in our review showed that AFEQT demonstrated strong positive evidence for internal consistency and content validity and AFQoL showed strong positive evidence for content validity. All other questionnaires failed to provide sufficient evidence to make a strong positive rating. Neither measurement error nor cross-cultural validity were appropriately assessed in any study and there were limited data for responsiveness, which we were only able to rate for AFEQT. Where study quality was poor, or ratings indeterminate, this does not mean that a particular questionnaire performed badly, only that the evidence was limited. For example, AFQoL, in addition to AFEQT, demonstrated strong positive evidence for content validity, an important measurement property that is the foundation of a good QoL questionnaire.

Refinements to AF PROMs are ongoing, and AFQLQ was recently updated to a second version, demonstrating good intra- and inter-observer reproducibility and internal consistency in 40 AF patients. [36] However, even with full information on psychometric properties, clinical validity requires further appraisal in large-scale AF populations, a review of which is beyond the scope of this methodological assessment. Apart from AFEQT [37], there is limited information about the minimally important change seen with these questionnaires.

With the growing burden of AF and the equivocal benefit of rate or rhythm-control strategies in improving mortality [6–9], there is increasing importance of QoL assessment. Over 34 different QoL questionnaires have been utilized in published AF studies [10] emphasizing the current lack of consensus on the best instrument to assess QoL. Our assessment of methodological quality suggests that reported results for the available instruments should be treated with caution and that further, more robust validation is required to determine if these measures are appropriate to assess QoL in clinical populations. However, adequate measurement properties are just one aspect of whether a QoL tool is useful in research and clinical practice. Cost, ease of administration, time taken and patient acceptability are also important considerations. [29] Future studies are clearly required that address the detectable change in scores, as well as the responsiveness of the questionnaire to changes in patient symptoms and management strategies in the clinical setting. One such study that is due to commence shorty is the RAte Control Therapy Evaluation in Atrial Fibrillation (RATE-AF) randomized trial. [38]

## Conclusion

Using the systematic COSMIN approach, we have identified clear deficiencies in the five available instruments for assessing disease-specific quality of life in patients with atrial fibrillation. The strongest performing tool was AFEQT, but further evidence for test-retest reliability, measurement error and responsiveness are required before recommending routine clinical implementation.

## Supporting Information

S1 Appendix Search strategy for Medline. (DOCX)

S2 Appendix PRISMA checklist. (PDF)

S1 Table COSMIN criteria for measurement properties. (DOCX)

S2 Table COSMIN criteria for data synthesis. (DOCX)

S3 Table Table of excluded full-text studies. (DOCX)

S4 Table Validity assessment. (DOCX)

S5 Table Reliability and responsiveness assessment. (DOCX)
