# Supplementary material for: Patient-Reported Outcomes for Quality of Life Assessment in Atrial Fibrillation: A Systematic Review of Measurement Properties
Source: PLoS One. 2016 Nov 1;11(11):e0165790. doi: 10.1371/journal.pone.0165790 (PMC5089715; doi:10.1371/journal.pone.0165790)
Supplement: S1 Table — (DOCX) [file pone.0165790.s003.docx]

## S1 Table: COSMIN criteria for measurement properties

| **Property** | **Rating^†^** | **Quality Criteria** |
| --- | --- | --- |
| **Reliability** | | |
| Internal consistency | **+** | Cronbach's alpha(s) ≥ 0.70 |
|  | **?** | Cronbach's alpha not determined or dimensionality unknown |
|  | **-** | Cronbach's alpha(s) < 0.70 |
| Test-retest reliability | **+** | ICC / weighted Kappa ≥ 0.70 OR Pearson’s r ≥ 0.80 |
|  | **?** | Neither ICC / weighted Kappa, nor Pearson’s r determined |
|  | **-** | ICC / weighted Kappa < 0.70 OR Pearson’s r < 0.80 |
| Measurement error | **+** | MIC > SDC OR MIC outside the LOA |
|  | **?** | MIC not defined |
|  | **-** | MIC ≤ SDC OR MIC equals or inside LOA |
| **Validity** | | |
| Content validity | **+** | All items are considered to be relevant for the construct to be measured, for the target population, and for the purpose of the measurement AND the questionnaire is considered to be comprehensive |
|  | **?** | Not enough information available |
|  | **-** | Not all items are considered to be relevant for the construct to be measured, for the target population, and for the purpose of the measurement OR the questionnaire is considered not to be comprehensive |
| Construct validity | | |
| - *Structural validity* | **+** | Factors should explain at least 50% of the variance |
|  | **?** | Explained variance not mentioned |
|  | **-** | Factors explain < 50% of the variance |
| - *Hypothesis testing* | **+** | Correlations with instruments measuring the same construct ≥ 0.50 OR at least 75% of the results are in accordance with the hypotheses AND correlations with related constructs are higher than with unrelated constructs |
|  | **?** | Solely correlations determined with unrelated constructs |
|  | **-** | Correlations with instruments measuring the same construct < 0.50 OR < 75% of the results are in accordance with the hypotheses OR correlations with related constructs are lower than with unrelated constructs |
| - *Cross-cultural validity* | **+** | No differences in factor structure OR no important DIF between language versions |
|  | **?** | Multiple group factor analysis not applied AND DIF not assessed |
|  | **-** | Differences in factor structure OR important DIF between language versions |
| Criterion validity | **+** | Convincing arguments that gold standard is “gold” AND correlation with gold standard ≥ 0.70 |
|  | **?** | No convincing arguments that gold standard is “gold” |
|  | **-** | Correlation with gold standard < 0.70 |
| **Responsiveness** | | |
| Responsiveness | **+** | Correlation with changes on instruments measuring the same construct ≥ 0.50 OR at least 75% of the results are in accordance with the hypotheses OR AUC ≥ 0.70 AND correlations with changes in related constructs are higher than with unrelated constructs |
|  | **?** | Solely correlations determined with unrelated constructs |
|  | **-** | Correlations with changes on instruments measuring the same construct < 0.50 OR < 75% of the results are in accordance with the hypotheses OR AUC < 0.70 OR correlations with changes in related constructs are lower than with unrelated constructs |

AUC, area under the curve; DIF, differential item functioning; ICC, intraclass correlation coefficient; LoA, limits of agreement; MIC, minimal important change; SDC, smallest detectable change. **^†^** + = positive rating; ? = indeterminate rating; - = negative rating. Table adapted from Terwee *et al*.[17]
