# Supplementary material for: Patient-Reported Outcomes for Quality of Life Assessment in Atrial Fibrillation: A Systematic Review of Measurement Properties
Source: PLoS One. 2016 Nov 1;11(11):e0165790. doi: 10.1371/journal.pone.0165790 (PMC5089715; doi:10.1371/journal.pone.0165790)
Supplement: S2 Table — (DOCX) [file pone.0165790.s004.docx]

## S2 Table: COSMIN criteria for data synthesis

| **Level** | **Rating^†^** | **Criteria** |
| --- | --- | --- |
| strong | **+++** or **- - -** | Consistent findings in multiple studies of good methodological quality OR in one study of excellent methodological quality |
| moderate | **++** or **- -** | Consistent findings in multiple studies of fair methodological quality OR in one study of good methodological quality |
| limited | **+** or **-** | One study of fair methodological quality |
| conflicting | **+/-** | Conflicting findings |
| unknown | ? | Only studies of poor methodological quality |

+ = positive rating; ? = indeterminate rating; - = negative rating.
