# Supplementary material for: Patient-Reported Outcomes for Quality of Life Assessment in Atrial Fibrillation: A Systematic Review of Measurement Properties
Source: PLoS One. 2016 Nov 1;11(11):e0165790. doi: 10.1371/journal.pone.0165790 (PMC5089715; doi:10.1371/journal.pone.0165790)
Supplement: S3 Table — (DOCX) [file pone.0165790.s005.docx]

S3 Table: Table of excluded full-text studies

| **First author, year** | **Full reference** | **Summary of study** | **Reason for exclusion** |
| --- | --- | --- | --- |
| Tsuneda, 2006 | Tsuneda, T., et al. (2006). "Rate control and quality of life in patients with permanent atrial fibrillation: The Quality of Life and Atrial Fibrillation (QOLAF) Study." Circulation Journal 70(8): 965-970. | Effectiveness of rate control agents on quality of life in AF. | Measurement properties of quality of life questionnaires were not assessed. |
| Reynolds, 2008 | Reynolds, M. R., et al. (2008). "Quality of life in atrial fibrillation: measurement tools and impact of interventions." Journal of Cardiovascular Electrophysiology 19(7): 762-768. | Reviews the designs, strengths and limitations of quality of life questionnaires in AF. | Review paper - no measurement properties were assessed. |
| Yamashita, 2011 | Yamashita, T., et al. (2011). "Randomized trial of angiotensin II-receptor blocker vs. dihydropiridine calcium channel blocker in the treatment of paroxysmal atrial fibrillation with hypertension (J-RHYTHM II Study)." Europace 13(4): 473-479. | Candesartan versus amlodipine in the treatment of paroxysmal AF associated with hypertension. | Quality of life questionnaire used as an endpoint, but methodological assessment was not performed. |
| Walfridsson, 2012 | Walfridsson, U., et al. (2012). "Development and validation of a new Arrhythmia-Specific questionnaire in Tachycardia and Arrhythmia (ASTA) with focus on symptom burden." Health and Quality of Life Outcomes 10(44). | Design and evaluation of a symptom checklist for patients with tachycardia or arrhythmia. | Symptom checklist rather than a quality of life questionnaire. |
| Dorian, 2013 | Dorian, P., et al. (2013). "Interpreting changes in quality of life in atrial fibrillation: How much change is meaningful?" American Heart Journal 166(2): 381-387. | Interpretation of changes in the AFEQT score. | No measurement properties on reliability or validity were assessed. |
| Yamamoto, 2014 | Yamamoto, M., et al. (2014). "Association between the quality of life and asymptomatic episodes of paroxysmal atrial fibrillation in the J-RHYTHM II study." Journal of Cardiology 64(1): 64-69. | Correlation of asymptomatic AF episodes with quality of life. | Measurement properties of quality of life questionnaires were not assessed. |
| Wynn, 2015 | Wynn, G. J., et al. (2015). "Quality-of-life benefits of catheter ablation of persistent atrial fibrillation: A reanalysis of data from the SARA study." Europace 17(2): 222-224. | Comparison of catheter ablation and antiarrhythmic drug therapy on quality of life. | Measurement properties of quality of life questionnaires were not assessed. |
