# Supplementary material for: Patient-Reported Outcomes for Quality of Life Assessment in Atrial Fibrillation: A Systematic Review of Measurement Properties
Source: PLoS One. 2016 Nov 1;11(11):e0165790. doi: 10.1371/journal.pone.0165790 (PMC5089715; doi:10.1371/journal.pone.0165790)
Supplement: S4 Table — (DOCX) [file pone.0165790.s006.docx]

S4 Table: Validity assessment

| **Instrument** | **Content validity** | **Criterion validity** | **Construct validity*** | |
| --- | --- | --- | --- | --- |
|  |  |  | **Structural validity** | **Hypothesis testing** |
| **AF6[24, 25]** | - Original questionnaire 7 items determined by AF expert nurse and most frequent patient-reported problems. | - All items at baseline compared to 3 levels of symptom severity from SCL questionnaire during known group validity assessment. - Distinguished between patients with different frequencies and severities of symptoms based on SCL. | - Initial 7-item questionnaire was divided into 2 domains. - Item 3 removed after factor analysis. Rasch analysis confirmed 6-item questionnaire was uni-dimensional. | - Discriminant and convergent validity assessed via Pearson correlation coefficient between AF6, SCL and SF-36. - Higher correlations between AF6 items to related domains on SCL and SF-36. |
| **AFEQT[20]** | - Developed from literature search of QoL in AF, followed by interviews with patients and experts. - Initial 117-item questionnaire given to 148 patients; afterwards items ↓ to 42-items. - Cognitive interviews with 12 patients to ensure readability & comprehension. - Revisions made; additional 12 patients interviewed again for comprehension. | - Correlated with SF-36. | - Factor analysis: 5 factors of 47 items, reduced to 39 items. 3 satisfaction items added. - Second factor analysis reduced to 3 domains of 18 items with 1 satisfaction item removed. - Final questionnaire: 20 items across 4 domains, including 2 satisfaction items. | - AFEQT: greater correlation with hypothesized similar domain on SF-36, AFSS and SCL, but lower correlation with hypothesized unrelated domains on the same questionnaires. - Known group validity was assessed and patients with severe AF symptoms scored a lower global score. |
| **AFQLQ[15, 16]** | **^†^** | **^†^** | **^†^** | **^†^** |
| **AFQoL[21, 22]** | - Literature search on AF plus other QoL questionnaires administered to AF patients. Domains created with help of AF experts. - Interviews with 17 AF patients; 286 expressions identified, reduced to 94 and with further expert involvement reduced to 40. - 40-item questionnaire given to pilot sample of 112 AF patients. | - Compared scores to NYHA class and patient self-perceived health status questionnaire. - Higher NYHA class and worse health status associated with poorer scores on AFQoL. | - Factor analysis: 2 factors (physical and psychological) explained 50.2% of the variance. - Rasch analysis reduced items from 27 to 7 in factor 1 and from 19 to 11 in factor 2. | - Spearman’s rank correlation for domain comparison. - Scores on the AF-QoL and SF-36 questionnaires at baseline visit correlated (>0.5) in all but one domain. - AF-QoL compared to SF-36 bodily pains domains r= 0.32. |
| **QLAF[23]** | - Validated against literature plus expert opinion (3 meetings with 8 specialists determined most important clinical manifestations). - Preliminary questionnaire formed; discussed with specialists for relevance. - Administered to a small group of patients during pilot phase (number not reported). | - Simultaneous administration of QLAF & SF-36. Global scores assessed for correlation. - Higher scores on SF-36 correlated with lower scores on QLAF; both represent better QoL. | **^†^** | - Convergent validity analyzed by comparison with SF-36. - Inverse correlation but same expression of QoL (higher score on SF-36 = lower score on QLAF); coefficient data not shown. |

* No assessment of cross-cultural validity was possible for any instrument.  ^†^ No data available for assessment. SCL, Toronto AF Symptoms Check List, AFSS, Atrial Fibrillation Severity Scale.
