# Supplementary material for: Patient-Reported Outcomes for Quality of Life Assessment in Atrial Fibrillation: A Systematic Review of Measurement Properties
Source: PLoS One. 2016 Nov 1;11(11):e0165790. doi: 10.1371/journal.pone.0165790 (PMC5089715; doi:10.1371/journal.pone.0165790)
Supplement: S5 Table — (DOCX) [file pone.0165790.s007.docx]

## S5 Table: Reliability and responsiveness assessment

| **Instrument** | **Reliability** | | | | **Responsiveness** | |
| --- | --- | --- | --- | --- | --- | --- |
|  | **Internal Consistency** | **Reliability** | | | **Method** | **Results** |
|  | **Cronbach’s alpha per domain/overall** | **Time interval** | **Method** | **Result** |  |  |
| **AF6[24, 25]** | α=0.81 | 12 days | ICC in the 9 non-responders to cardioversion. | Dyspnea at rest: ICC=0.82; Dyspnea on exertion: ICC=0.88; Limitations in daily life due to AF: ICC=0.19; Discomfort due to AF: ICC=0.04; Fatigue due to AF: ICC=0.39; Anxiety due to AF: ICC=0.93. | Effect Size was calculated in the overall cohort, in responders and non-responders. | Effect size: Responders: 0.10 to 0.85; Non-responders: -0.23 to 0.34. Effect size was larger per item in responders. |
| **AFEQT[20]** | α>0.88 | 1 month | ICC in 43 stable AF patients. | ICC=0.8. | Effect size of changes in AFEQT global score between baseline and follow up, in intervention and stable groups. | Effect size: Stable group=0.2; Medication group=0.5; Ablation group=1.1. |
| **AFQLQ[15, 16]** | Domain 1: α=0.78; Domain 2: α=0.81; Domain 3: α=0.89 | 121 days | Spearman’s rank correlation coefficient per question. | r>0.5 for all besides domain 3, question 5, 11, 13, 14. | Spearman rank correlation coefficient determined between AFQLQ scores determined at baseline and follow-up. | Type and frequency of symptoms: r=0.75; Severity of symptoms: r=0.75; Mental aspects/everyday life limitations: r=0.76. |
| **AFQoL[21, 22]**^†^ | α=0.92 | 1 month | Pearson's reliability during Rasch analysis. ICC in 84 stable AF patients. | Overall: Psychological (AFQoL-7)=0.82; physical (AFQoL-11)= 0.82. ICC>0.80 (actual values not reported). | Effect size AFQoL score at baseline and follow-up in those receiving a new therapeutic intervention compared with patient self-perceived health status questionnaire administered at the same time. | Effect size: Overall AFQoL=1.06 among AF intervention patients reporting an improvement in QoL as determined by the patient perceived health status questionnaire. |
| **QLAF[23]** | Inter-rater: α=0.98; Intra-rater: α=0.96* | ^‡^ | Inter and intra observer ICC. | Inter-rater: ICC=0.95; Intra-rater: ICC= 0.91. | Significant differences between changes in QLAF domain scores during the follow-up period. | Significant differences between changes domains and global score after 12-month follow-up. |

ICC, intraclass correlation coefficient. ^†^ Reliability assessed differently in the two studies. ^‡^ Not specified.
